# Resistance to Cry Intensive Sleep Intervention in Young Children: Are We Ignoring Children’s Cries or Parental Concerns?

**DOI:** 10.3390/children3020008

**Published:** 2016-05-10

**Authors:** Sarah Blunden, Hayley Etherton, Yvonne Hauck

**Affiliations:** 1 Appleton Institute Central Queensland University, Adelaide, South Australia 5043, Australia; h.etherton@cqu.edu.au; 2 School of Nursing, Midwifery & Paramedicine, Curtin University, Bentley, Western Australia 6102, Australia; Y.Hauck@curtin.edu.au

**Keywords:** child sleep problems, extinction, sleep intervention, crying

## Abstract

The majority of behavioural sleep interventions for young children (defined as 5 years of age or less) involve extinction procedures where parents must ignore their child’s cries for a period. Many parents have difficulties implementing and maintaining these procedures, leading to attrition, non-compliance and treatment avoidance. Yet the reasons for these methods being difficult to implement for parents have not been well understood or addressed in the literature. In fact, they are being ignored. We discuss that understanding and addressing parental concerns may enable better targeted sleep interventions.

## 1. Introduction

Studies estimate approximately one-third of parents identifies their child as having a sleep problem [1,2] because their child depends on parental assistance for initiating and reinitiating sleep overnight. Parents’ sleep disruption increases stress, anxiety, depression, and even the risk of child abuse [3] while reducing coping ability [1,4,5] and general wellbeing. Families often seek help from general practitioners, pediatricians, psychologists and other health professionals [6] who base their recommended sleep interventions on the current evidence.

Standard behavioural sleep intervention research studies have been reviewed for under five-year-olds [1] and were grouped into four interventions: extinction (unmodified extinction, graduated extinction and extinction with parent presence); positive routines/faded bedtime with response cost; scheduled awakening and parent education/prevention. Overall, the evidence suggests that behavioural sleep treatments are effective with 94% of treatments demonstrating clinically significant change. The majority of studies in this domain (81%) report trials in extinction-based sleep interventions and this is replicated in popular literature with 61% of parent sleep advice books endorsing extinction interventions [7]. Extinction interventions are dominating treatment discussion and practice which is understandable given their success [1].

Extinction sleep interventions are based on behaviour theory’s principle of extinction which proposes that a behavioural response is maintained or strengthened by the presence of a reinforcer [8]. For example, in the case of child sleep problems a child’s overnight crying and protesting behaviour is responded to by a parent (behavioural response) and this ‘maintains’ the continuation of the unwanted behaviour (crying). Extinction sleep interventions aim to improve sleep by removing parent attention to the child during sleep times to eliminate their night-time crying [9]. The use of extinction aims to create conditions for the child to learn to re-initiate sleep without parental assistance. Three extinction protocols are commonly used. During unmodified extinction (“cry it out”), first used in 1959 [10], the parent will ignore all the child’s calling, crying, protesting or tantrums until a set wake time in the morning. The exception is if parents believe the child is ill, hurt or in danger. In graduated extinction (“controlled crying”) parents periodically ignore the child’s crying for set periods of time, the length of which can vary between protocols, until the child settles alone. Finally, in extinction with parental presence (“camping out”), a parent remains in the child’s room while the child falls asleep, progressively moving further away each night until they do not remain with the child at all, and may choose to then use unmodified extinction or graduated extinction if necessary. 

Extinction-based interventions are reported to be successful in reducing night time crying and sleep disruption for infant and parents and thereby improving subsequent daytime mood and performance outcomes of parents [1,4,5]. Despite their efficacy in reducing night waking, parental resistance to extinction sleep interventions has been reported. Several studies [9,11,12,13,14,15,16] and reviews [1,17,18] have cited parental resistance as one of the difficulties in compliance, success and uptake of extinction interventions. 

It has been suggested [15,19] that this resistance is partly due to the post-extinction bursts (the reappearance of a previously extinguished behaviour, for example an initial increase in crying before it is ‘extinguished’) which are common and must also be ignored completely to prevent reinforcement. However, some parents are unable to withstand the stress of ignoring such intense distress and demands from their child for the length of time required for this method to work, which can be several nights to weeks. Indeed it is unclear whether parents are instructed to expect this post extinction burst and if not, may be excused for thinking that the intervention is “not working”, which subsequently leads them to stop. Parents may resist extinction interventions because they are too traumatic for them, contradict their beliefs about child-rearing, or because they are perceived to be impractical [20].

Whilst many studies cited above have reported that parents find ignoring such intense distress and demands from their child difficult, how much this contributes to attrition and non-compliance and can be accounted for by parent resistance to the intervention is rarely reported. It is therefore difficult to extrapolate reasons from the literature. Seymour, Bayfield, Brock and During [21] reported 5% of parents did not implement their intervention program and a further 2% could not be contacted after the initial interview. Tse and Hall [20] reported “objections to treatments” and “difficulty in completion” while Friman and colleagues [22] reported parental preferences for gentler methods of limit setting (such as the “bedtime pass” method) compared to ignoring. An Australian randomised control trial (RCT) of the two modified extinction interventions, graduated extinction and parental presence, reported 28% (*n* = 65) of eligible and contactable parents declined to participate [5]. About half of these (*n* = 33, 14%) declined because they reported only a mild sleep problem, but it is unclear what the remaining reasons were and how many parents declined to participate due to resisting the intervention. Owens *et al*. [15] noted a lack of parental acceptance of an intervention, but did not discuss the reasons for this resistance in detail. Additional papers have also mentioned parental resistance [3,15,23,24], without reference to detailed information.

However, some researchers have reported specific parental concerns. Rickert and Johnson [11] reported a 10% attrition rate specifically due to a refusal to ignore crying. Furthermore, additional parents had responded to the recruitment ad, but refused to allow a home visit and interview because they may have been allocated to the ignoring condition. Tse and Hall [20] reported that approximately 10% of their sample could not be contacted due to dropping out of their extinction study following “ethical concerns” although exactly what these concerns refer to were not reported. In Reid, Walter and O’Leary’s [12] study, 22 of 71 (33%) parents of young children elected not to participate in extinction interventions. Finally, Hiscock [25] described another study (unpublished) which planned to investigate the efficacy of an extinction based sleep intervention and relationships with stress and attachment. In that study, optimal sample recruitment was compromised because enough parents reported opposition to those behavioural sleep interventions.

Despite these reports, specific parental concerns in this domain have rarely been the subject of specific investigation, so reasons for resisting or stopping extinction sleep interventions remain unclear. Only two published studies have reported specific parental concerns. The first cited that 70% of parents did not undertake extinction methods, or started and then stopped for reasons reported as largely the perceived emotional or stressful impact on parents and child [26]. The second reported similar findings with 60% [27] of surveyed parents not using graduated extinction methods or using it and stopping while citing comparable reasons.

In order to better understand the impact of extinction methods on parental and child distress, Hiscock *et al*. have undertaken several studies to evaluate potential impacts of extinction on the child’s wellbeing and behaviour, parental mood and stress levels, and parent/child attachment over several time points and up to six years after the intervention. In that final study [28] results showed that there were no significant differences between those who had undertaken extinction sleep interventions and those who had not done so on any measure, suggesting no negative (nor positive) impact on parent or child in the long term. Whilst this is reassuring, there are competing arguments in another study [29] that leaving an infant to cry unattended in the short term caused an immediate rise in the stress hormone cortisol, which remained high in the days after the infants had stopped crying. The authors interpreted this as evidence of the immediate and significant impact of extinction interventions on an infants’ stress level although with no normative data on infant cortisol levels this conclusion may be preliminary.

Evidence therefore is not only scarce but also conflicting, and so further research is required to understand how extinction interventions impact children. Likewise there is a paucity of research understanding parents’ willingness to undertake extinction interventions. The limited evidence available suggests for some, extinction interventions cause significant stress in the immediate short term, so much so that, despite evidence indicating effectiveness, many parents may be refusing to undertake extinction interventions. Sleep problems and their subsequent effects in those cases may remain untreated and consequential, unless those parents can be offered alternative options such as a non-extinction based treatment. Indeed, non-extinction based sleep interventions are available, published and successful [30,31,32].

So it appears that *despite* their success in reducing night wakings, some parents are resistant to extinction sleep interventions for their child. Parents need to be informed of the available evidence-based options for their child’s sleep problem to enable them to make an informed decision. Health professionals need to be informed of the alternative evidence-based interventions so intervention options presented to parents can be choice rich.

It seems that both parental concerns and infant crying are being ignored.
